# Clinical validation of perfusion imaging with pulmonary function test data using Voronoi-based discretization

**DOI:** 10.1088/1361-6560/ae4669

**Published:** 2026-03-03

**Authors:** Jorge Cisneros, Caleb J Herrera, Yi-Kuan Liu, Lisa V Du, Yevgeniy Vinogradskiy, Richard Castillo, Girish Nair, Edward Castillo

**Affiliations:** 1 University of Texas at Austin, 107 W Dean Keeton St, Austin, TX, United States of America; 2 University of Texas Southwestern Medical Center, 5323 Harry Hines Blvd, Dallas, TX, United States of America; 3 Thomas Jefferson University, 4201 Henry Ave, Philadelphia, PA, United States of America; 4 Emory University School of Medicine, 2015 Uppergate Dr, Atlanta, GA, United States of America; 5 University of Kentucky College of Medicine, 780 Rose St MN 150, Lexington, KY 40506, United States of America

**Keywords:** pulmonary function test, SPECT imaging, CT-based functional prediction, Voronoi diagram

## Abstract

*Objective.* Accurate lung function assessment is essential for diagnosing and managing diseases like chronic obstructive pulmonary disorder, pulmonary emboli, and lung cancer. Single-photon emission computed tomography (SPECT) provides valuable 3D functional imaging of ventilation and perfusion, but is limited by low spatial resolution, availability, additional radiation, and cost. Alternative methods, including CT-based perfusion (CT-P) and deep learning models, require large datasets to validate results that are often scarce. Pulmonary function tests (PFTs) offer rapid and noninvasive global lung function measures and are clinically widely used. While ventilation correlates well with PFTs, perfusion imaging presents challenges due to complex blood flow and difficulty summarizing 3D data into one value. Additionally, commonly employed percentile scaling removes absolute quantitative information, complicating interpretation. *Approach.* We propose a framework leveraging lung discretizations based on Voronoi diagrams to capture local spatial information from raw-valued and percentile-scaled perfusion maps (SPECT and CT-P). We compute hierarchical descriptive statistics at 3 levels (intra-subvolume, inter-subvolume, left-right lungs) to derive one global value per patient. *Main results.* Across PFT measures of diffusing capacity of lungs for carbon monoxide, forced expiratory volume after one second (FEV1), and FEV1/forced vital capacity, we find that discretizing perfusion maps into Voronoi subvolumes always yields stronger Spearman correlations than not discretizing. Specifically, our approach demonstrates strong correlations of $0.636 \unicode{x2A7D} \rho \unicode{x2A7D} 0.843$ (*P* < 0.005) for raw-valued (SPECT and CT-P) maps, $0.590 \unicode{x2A7D} \rho \unicode{x2A7D} 0.789$ (*P* < 0.005) for percentile-scaled maps, and reliably distinguishes normal from abnormal lung function via logistic regression analysis ($0.865 \unicode{x2A7D} \mathrm{AUC} \unicode{x2A7D} 0.937$ for raw-valued maps, $0.877 \unicode{x2A7D} \mathrm{AUC} \unicode{x2A7D} 0.933$ for percentile-scaled maps). *Significance.* This framework bridges regional perfusion imaging and global pulmonary function assessment, enabling meaningful quantitative comparisons between SPECT and CT-P maps. By preserving local spatial variability, the method offers a noninvasive tool for integrating imaging and physiological data, paving the way toward broader clinical and AI-driven applications in lung function evaluation.

## 1. Introduction

Assessing lung function accurately and cost-effectively are key in managing patients with lung diseases, like chronic obstructive pulmonary disorder (COPD), pulmonary embolism, and lung cancer. Single-photon emission computed tomography (SPECT) is a nuclear medicine imaging modality that provides 3D spatial information on the functional status of the lungs by detecting gamma rays emitted from inhaled or intravenously-administered radiotracers (Bourhis *et al* 2020, Bang *et al* 2024, Mirza and Hashmi 2024). SPECT imaging can visualize physiological processes such as ventilation (air flow) or perfusion (blood flow) after registering to a lung CT scan, allowing physicians to directly analyze regions of low functionality, diagnose diseases such as pulmonary embolism, COPD, emphysema, and interstitial lung disease, and appropriately plan radiotherapy treatments for lung cancer patients (Bajc and Jonson 2011, Bateman 2012, Bajc and Lindqvist 2019, 2025, Iqbal *et al* 2023).

Despite its ability to provide valuable functional information, SPECT imaging has relatively low spatial resolution, is susceptible to attenuation artifacts, carries high cost, is not widely available, and increases patient radiation dosage (Elojeimy *et al* 2016, Schreuder *et al* 2019, Yandrapalli and Puckett 2025). These factors motivate the development of alternative approaches to produce functional ventilation and perfusion maps. Non-contrast CT avoids the use of radiopharmaceuticals and contrast agents and offers high-quality structural information, but provides minimal functional information. Even so, non-contrast CT has become the standard of care for lung cancer patients, where these scans are routinely available for patients undergoing lung cancer screening, staging, or follow-up (Wang *et al* 2019, Chen *et al* 2021). While contrast-enhanced modalities such as SPECT or CT pulmonary angiography are routinely used to evaluate suspected pulmonary embolism, non-contrast CT offers a safer and more practical option for patients with renal impairment or contrast allergies, motivating the development of contrast-free perfusion surrogates (Wood 2002, Hogan *et al* 2019). Physics-based approaches initially estimated lung ventilation (CT-V) from paired end-inspiration (inhale) and end-expiration (exhale) non-contrast CT scans by analyzing regional changes in Hounsfield units (Simon 2000). Building on this, the Integrated Jacobian Formulation enhanced robustness by modeling volume changes and was further extended to estimate pulmonary perfusion (CT-P) through regional mass differences between spatially aligned inhale and exhale non-contrast CT volumes (Castillo *et al* 2019, Castillo 2021). Despite their rigor, these approaches depend heavily on accurate lung segmentation and deformable registration, making them sensitive to noise and artifacts. With the rise of AI, deep learning approaches have recently been proposed to generate synthetic pulmonary perfusion images from clinical non-contrast CT for patients undergoing radiotherapy (Porter *et al* 2021, Ren 2022, Liu *et al* 2025). Many of these approaches, however, lack extensive validation due to the scarcity and restriction of large paired datasets of non-contrast CT and SPECT perfusion images. One promising alternative is to examine relationships between scores derived from SPECT imaging and scores derived from non-contrast CT-based methods with scores from pulmonary function tests (PFTs).

PFTs are quick, low-cost, and noninvasive exams that reveal global ventilation and perfusion information about lung function and are critical in patients’ diagnosis (Schneider *et al* 2009, Ruppel and Enright 2012, Sumphao-Ngern *et al* 2015, Li *et al* 2019, Ponce *et al* 2025). PFTs have been well-established in clinical practice since the 1980s, when standardization efforts and landmark studies cemented their role in respiratory disease diagnosis (Fletcher and Peto 1977, Miller 2005, Tojo 2014). PFT assesses ventilation via spirometry, measuring critical parameters like forced expiratory volume after one second (FEV1), forced vital capacity (FVC), and the ratio between the two (FEV1/FVC). In particular, perfusion is assessed via measuring the efficiency of gas exchange across the alveolar-capillary membrane, quantified by the diffusing capacity of lungs for carbon monoxide (DLCO). Several studies have examined correlations between SPECT and CT-based ventilation maps with PFT data, finding strong relationships with both FEV1 and FEV1/FVC (Yamamoto 2014, Brennan 2015, Miller 2023). The more complex physiology of pulmonary blood flow has resulted in fewer investigations linking perfusion maps with PFT data (Roux *et al* 2015, Schiwek 2022).

**Figure 1.**
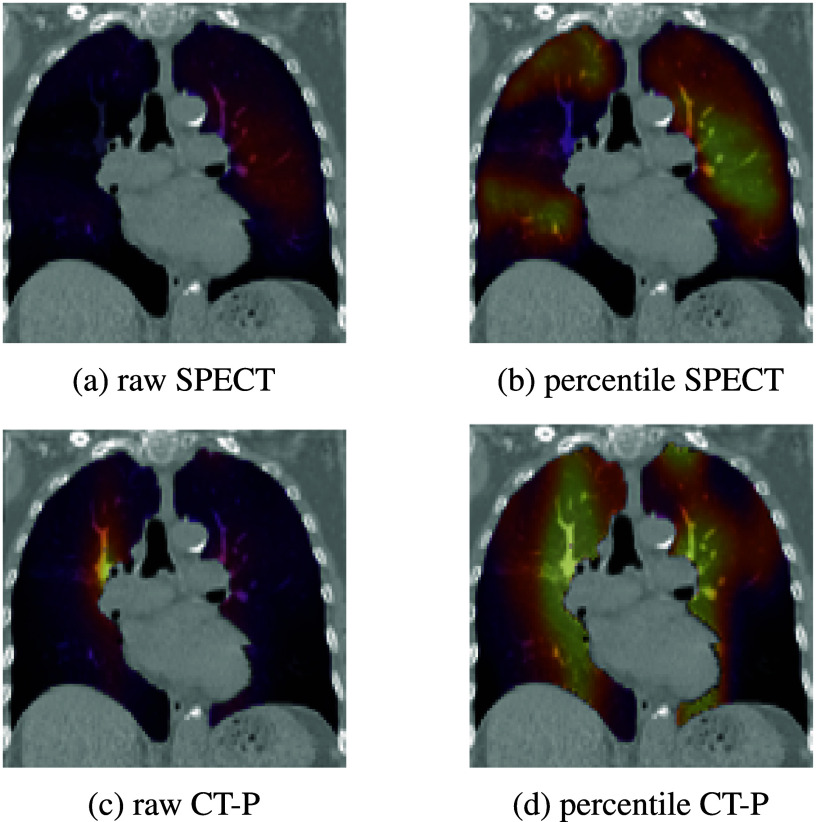
Coronal views of a SPECT perfusion map (top row) of raw values and percentiles alongside a CT-P estimate (bottom row) of SPECT-like raw values and percentiles, which has a Spearman correlation *ρ* = 0.36 with the SPECT image. Brighter colors correspond to high-perfusion regions.

A key challenge lies in projecting the 3D perfusion image into a single interpretable value that reflects the degree and heterogeneity of perfusion and can be meaningfully compared with PFT measurements. Furthermore, it is standard practice to normalize raw SPECT values to percentiles in order to reduce variability from patient size, injected dose, and acquisition settings (Brahim *et al* 2015, Schmitz-Steinkrüger *et al* 2020, Apostolova *et al* 2023, Seoni *et al* 2024). However, percentile scaling removes absolute quantitative information, which limits assessment of global functionality and explicit comparison with quantitative data. Furthermore, as AI-based methods gain traction, training deep learning models on normalized images has become increasingly common (Choi *et al* 2021, Kawakubo 2024, Kim *et al* 2024, Salimi 2025). Due to the scarcity of large paired datasets, models often normalize and predict perfusion maps for the left and right lungs independently, effectively doubling the training data while severing the global physiological relationship between the lungs (Liu *et al* 2025). Extracting a meaningful summary statistic is further complicated with these percentile-scaled left-right perfusion maps, as conventional quantitative metrics are not directly applicable. Figure 1 depicts a SPECT perfusion scan and CT-P prediction for the same patient (rows), in both raw values and percentiles (columns), where the latter provides a clearer distinction between regions of low and high perfusion.

We hypothesize that local spatial information within a percentile image retains valuable functional variability, albeit in relative terms. The purpose of this study is to develop methods that extract local perfusion information and condense it into a single score for a patient. To validate this approach, we treat SPECT perfusion as the clinical reference standard and compare it with PFT data, while also evaluating CT-P to determine whether it produces comparable results, thereby supporting the reliability of non-contrast, computationally derived perfusion estimates. Through the use of Voronoi diagrams, we create subvolumes within each left (L) and right (R) lung, followed by the application of descriptive statistics at the *intra*-subvolume level, *inter*-subvolume level, and the LR-lung level in order to extract one summary statistic. We find exceptional Spearman correlations between PFT data and derived values from raw and percentile SPECT and physics-based CT-P perfusion maps. Using logistic regression analysis, we further demonstrate that these summary values robustly predict normal versus abnormal lung function as defined by PFT measurements.

## 2. Methods

### 2.1. Dataset with perfusion imaging and PFT data

In this study, we rely on a cohort of 20 patients from the Beaumont Health System (Royal Oak, MI, IRB # 2016-037) (Vinogradskiy 2022) that yields a retrospective internal dataset of 36 imaging cases, each with a SPECT perfusion map, attenuation correction SPECT/CT, 4DCT imaging, and PFT data (only 33 of the cases have DLCO data). Most patients received thoracic interventions, like surgery, chemotherapy, or radiation, so pre- and post-treatment imaging was available. Most patients were diagnosed with non-small cell lung cancer (various stages), diagnosed with COPD, or were current or former smokers at the time of imaging.

PFT metrics of DLCO (%), FEV1 (%), FVC (%), and FEV1/FVC were expressed as percent-predicted values based on healthy individuals of similar height, age, and sex, with lower values indicating poorer lung function. When only raw values were available, they were converted to percent predicted using the lower limit of normal from the Global Lung Function Initiative calculators (Stanojevic *et al* 2017).

SPECT perfusion imaging was performed on a dual-head Siemens Symbia SPECT/CT (Siemens Medical Solutions, USA) with a high-resolution parallel-hole collimator and 15% energy window at 140 keV. After intravenous injection of 4.0 mCi 99mTc-MAA (Lantheus Medical Imaging, USA), patients were scanned supine during tidal breathing. Data was acquired in 6^∘^ steps over 360^∘^ (25 s/projection, $ < 30$ min total). Low-dose CT for attenuation correction was obtained at 130 kVp and 75–100 mAs (weight-dependent). Final reconstructed SPECT images were 64 × 64 per 2D slice image with resolution 6.00 mm × 6.00 mm × 2.00 mm.

4DCT imaging captures a time-series of ten 3D CT scans over the breathing cycle, providing a ‘movie’ of pulmonary anatomy during free breathing (Vedam *et al* 2002, Pan *et al* 2004, Zeng *et al* 2008). The 4DCT sets were acquired on a Philips Brilliance Big Bore scanner (v3.6.7) with a bellows system, using 120 kVp and 599 mAs. The final images were 512 × 512 × [77–160] with resolution [0.98–1.30] mm × [0.98–1.30] mm × 3.00 mm.

CT-based perfusion (CT-P) methods compute voxel-wise blood mass changes solely from dynamic non-contrast CT scans to yield pulmonary perfusion estimates like those akin to SPECT imaging. For this study, the inhale and exhale 4DCT phases are automatically extracted and segmented using the TriSwinUNETR model by Nomura (2025). These paired phases are then processed through a mass conservation framework: the segmented lungs independently undergo deformable image registration and Jacobian computation to capture local volume changes, where the voxel-wise perfusion is formulated as a constrained linear least-squares solution based on subregional mean mass changes. Castillo (2021) showed the resulting CT-P images provide spatial maps of magnitude mass change that serve as surrogates for perfusion, with an average correlation coefficient of 0.54 with SPECT perfusion. Finally, low-resolution SPECT perfusion maps are affine-aligned to the inhale CT image to enable direct comparison with CT-P predictions.

To create percentile images, we independently map raw values from the left and right lungs to their empirical percentiles, assigning each voxel a value from 0 to 1 based on its relative intensity within that lung. In summary, each case in the internal dataset contains four perfusion map types: a SPECT perfusion image of raw values (denoted as ‘raw SPECT’) and its corresponding percentile-scaled image (‘percentile SPECT’), and a CT-P perfusion prediction with SPECT-like raw values (‘raw CT-P’) and its corresponding percentile-scaled image (‘percentile CT-P’), as shown in figure 1.

### 2.2. Voronoi diagrams

To capture local spatial functionality in perfusion maps, we discretize each lung into subvolumes after resampling to isotropic spacing. Metrics are computed within each subvolume to assess local variation (intra-subvolume), compared across all subvolumes within the lung to capture broader regional differences (inter-subvolume), and finally evaluated between the left and right lungs to account for asymmetries. This hierarchical approach integrates information across multiple scales, ultimately producing a single summary statistic that reflects the overall variability in regional perfusion.

**Figure 2.**
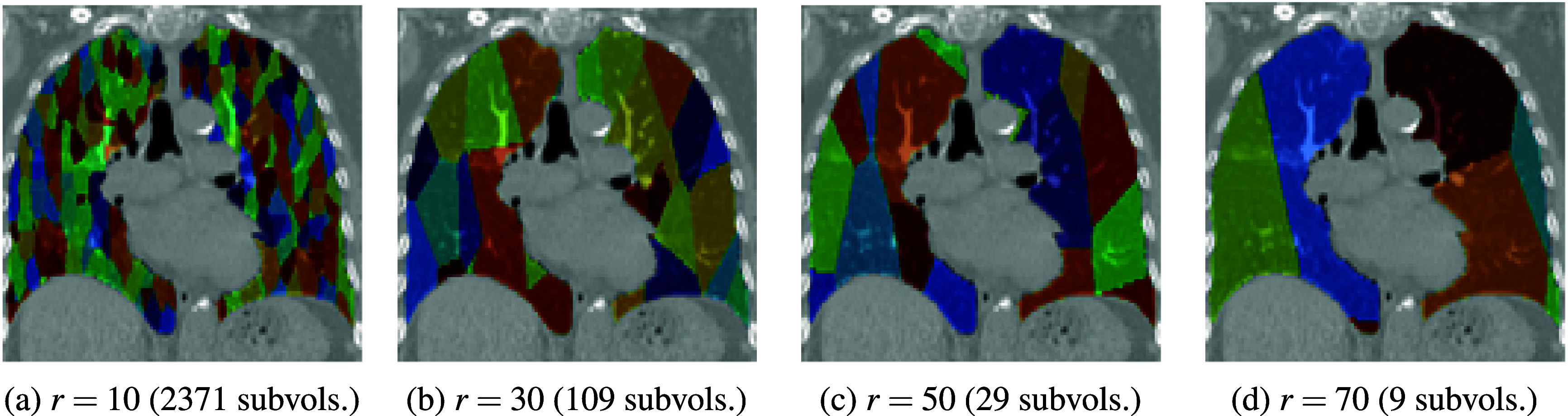
Coronal views of Voronoi diagrams at various radii. Subvolume counts are averaged over random seed point placements for the same radius.

Rather than relying on arbitrary grids, Voronoi diagrams provide a geometry-driven way to discretize space according to proximity, ensuring that each subvolume is localized and adaptive to seed placement (Aurenhammer and Klein 1996). This approach is well known, optimized, and has been successfully applied across biomedical applications and imaging modalities, like segmentation tasks in retinal imaging and confocal microscopy and registration tasks in 4DCT imaging (Bertin *et al* 1993, Fukuyama *et al* 2017, Huang *et al* 2018, Castillo 2019, 2021, Castillo *et al* 2019, Jalili *et al* 2020). In our case, we discretize each left and right lung using Voronoi diagrams to create subvolumes that naturally capture local anatomical neighborhoods around seed points. By generating isotropic seed points with Poisson disk sampling using Bridson’s algorithm (Bridson 2007), we ensure they are uniformly distributed with a minimum spacing *r*, producing well-separated, spatially contiguous subvolumes that enable an interpretable analysis of local perfusion patterns. Unlike random partitions, grids, or quadtrees, Voronoi cells naturally conform to irregular lung boundaries, preserve local neighborhood structure, and allow explicit control over subvolume size, ensuring consistent spatial resolution across subjects. In contrast to orientation- or intensity-driven methods, this approach is robust to image noise and avoids arbitrary spatial bias. Moreover, applying Voronoi-based discretization hierarchically to percentile-scaled images represents a novel strategy for extracting meaningful information. Figure 2 shows Voronoi diagrams at various radii, with subvolume counts averaged to account for random seed locations. Note that as $r \rightarrow 1$, the subvolumes approach the size of individual voxels and as $r \rightarrow \infty$ (practically, all *r* > 100), they approach the size of the full lung. At both extremes, descriptive metrics are meaningless, so we perform a grid search of $r \in [10, 90]$ to find the radius that produces the strongest correlation across an array of metrics at each level for our cohort.

### 2.3. Extracting a global statistic from perfusion maps

No established metric exists for correlating perfusion functional maps with PFT data, particularly when these maps are computed independently for left and right lungs, expressed as percentiles, or partitioned into discrete regions. To address this, we propose a brute-force search within a hierarchal structure to identify optimal combinations of metrics that best correlate with PFT outcomes for each of the four perfusion map types.

**Figure 3.**
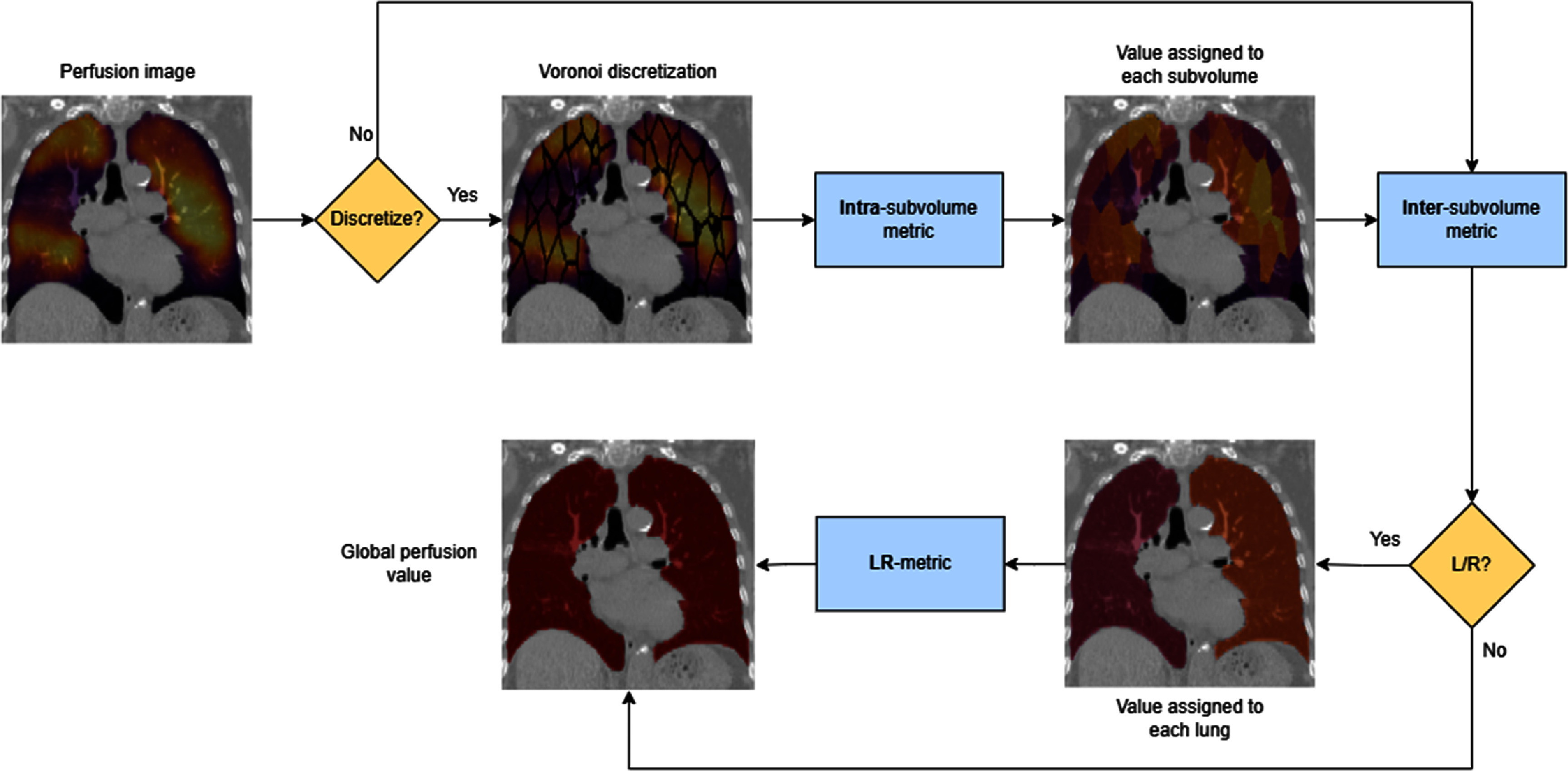
Flowchart illustrating the hierarchical intra-subvolume, inter-subvolume, and LR lung metrics on a perfusion map, whether it is discretized or LR split.

Our approach computes metrics in up to three hierarchical levels, depending on how the perfusion data is processed figure 3 illustrates these processing pathways. We illustrate this hierarchy using the most comprehensive case: a perfusion map of either raw values or percentiles that is split into independent left and right lungs, each discretized into Voronoi subvolumes of radius *r* (denoted as ‘Voronoi L/R’).

At the first level (intra-subvolume), we summarize the voxel intensities within each Voronoi subvolume using the following descriptive statistics: minimum, maximum, median, mean, coefficient of variation (CoV), 25th percentile, 75th percentile, skewness, and mode/median ratio. CoV is defined as the ratio of the standard deviation to the median. Skewness quantifies asymmetry in the distribution as the normalized third central moment, where positive values indicate a tail toward higher intensities (Zwillinger and Kokoska 2000). The mode is estimated from a histogram as the midpoint of the bin with the highest count. After this step, each subvolume is characterized by a single value for each metric.

At the second level (inter-subvolume), we aggregate the intra-subvolume values across all subvolumes within each lung by computing: *P_N_*, CoV, mode/median ratio, median, 25th percentile, 75th percentile, and skewness. The metric *P_N_* represents the percentage of lung volume with perfusion at or below $N\%$ of the max value across all subvolumes, analogous to ventilation defect metrics described by Yamamoto (2014) and Brennan (2015). We perform a grid search over $N \in [5\%, 95\%]$. After this step, each lung is characterized by a single value for each combination of intra- and inter-subvolume metrics.

At the third and final level (left-right combination) for this example, we merge the left and right lung values into a single patient-level score using one of the following operations: minimum, maximum, average, weighted average by lung volume, weighted average by lung perfusion values, value of the largest lung by volume, value of the smallest lung by volume, value of the lung with most perfusion, or value of the lung with the least perfusion. This final level yields the global perfusion score to correlate with PFT data.

Not all processing scenarios require all three levels. When lungs are analyzed together without discretization (‘Full L+R’), only inter-subvolume statistics are applied directly to the combined lung volume. When lungs are analyzed together with Voronoi discretization (‘Voronoi L+R’), intra-subvolume statistics are followed by inter-subvolume statistics, with no left-right combination needed. When lungs are analyzed separately without discretization (‘Full L/R’), inter-subvolume statistics are computed for each lung and then combined at the left-right level (see figure 3 for a visual overview).

Because the Voronoi seeding algorithm is stochastic, we average the resulting global scores across five independent discretizations of the same radius to ensure stability. Although computing multiple metrics across the several levels can be computationally intensive, the independence of metrics and discretization allows for straightforward parallelization, greatly reducing the processing burden.

### 2.4. Correlation and utility with PFT data

All combinations of intra-subvolume, inter-subvolume, and LR metrics are computed for each case of the four perfusion map types (raw SPECT, percentile SPECT, raw CT-P, percentile CT-P) in the four scenarios (Full L+R, Full L/R, Voronoi L+R, Voronoi L/R). Then, each combination is correlated with DLCO (%), FEV1 (%), FVC (%), and FEV1/FVC data. To assess the strength and direction of these associations, we employ Spearman’s rank correlation coefficient *ρ*, which is well-suited for capturing monotonic relationships without assuming linearity or normality (Spearman 1904). The combination with the largest Spearman coefficient is selected as the best performing global summary statistic for that perfusion map type.

To assess whether the perfusion imaging-derived metrics were associated with clinically meaningful impairment, the PFT dataset is split into 2 groups: normal and abnormal lung function. Reflecting physicians’ tendency to err on the side of caution, we applied a conservative rationale for clinical relevance, labeling a case with abnormal function if DLCO $ < 60\%$, FEV1 $ < 70\%$, or FEV1/FVC $ < 70\%$ (Pellegrino 2005, Ponce *et al* 2025). Only when all three parameters exceeded the thresholds was a case deemed normal. For each of the four perfusion map types, we use logistic regression with leave-one-out cross-validation to test whether the top five global perfusion statistics across all PFT measures can discriminate lung function. To identify the most predictive combination of top global statistics, we perform a brute-force search while measuring pairwise collinearity and full multicollinearity for each global statistic’s variance inflation factor (VIF). We also present a cross-group VIF multicollinearity statistic to address the expected collinearity between metrics of the same group, simply avoiding computing collinearity between metrics which are meant to correlate with the same PFT parameter. Predictive performance is assessed using area under the curve (AUC), receiver operating characteristic (ROC) analyses, *F*1 scores, and accuracies.

## 3. Results

### 3.1. Correlation with PFT

We correlated the hierarchical metrics derived from raw-valued and percentile-scaled SPECT and CT-P maps with PFT measures across four scenarios of discretization and left/right lung independence. All computations were performed using laptop-grade CPU resources, without requiring specialized hardware such as GPUs. The CT-P method and hierarchical pipeline are software-agnostic and are not tied to a specific computing platform. On average, generating one CT-P map from 4DCT inhale/exhale pairs required ∼ 200 s, while processing any perfusion map type through the full hierarchical pipeline to obtain its perfusion score required less than 1 s.

**Figure 4.**
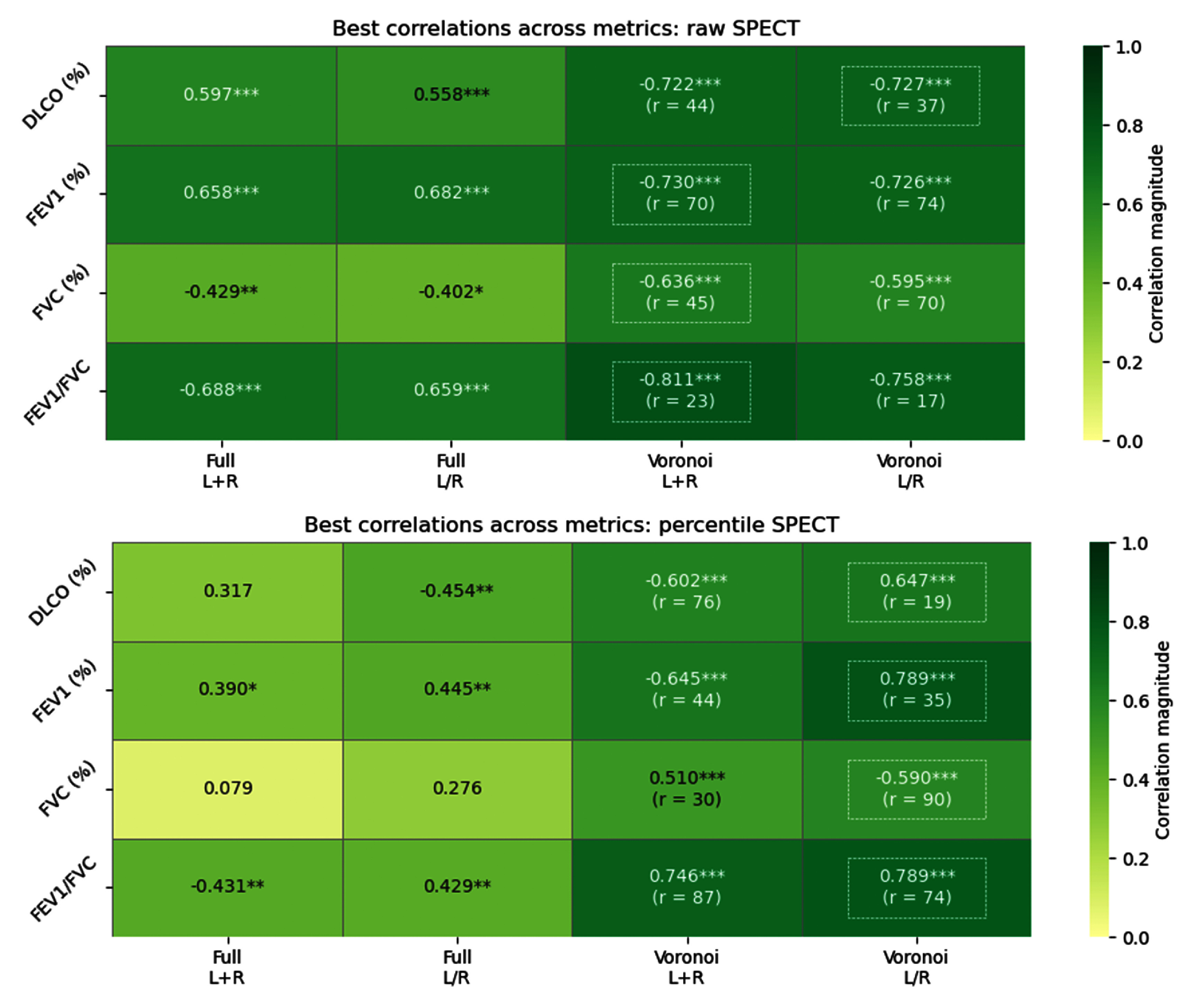
Spearman correlations for the best-performing hierarchical metrics on SPECT perfusion maps with raw values (top) and percentiles (bottom). ‘Full’ denotes the perfusion map was not discretized, while ‘Voronoi’ denotes the map was discretized with the given radius. ‘L+R’ denotes the lungs are treated together, while ‘L/R’ denotes the lungs are treated separately. The strongest correlation for each PFT measure is highlighted by a white dashed box. Statistical significances: *P < 0.05, **P < 0.01, ***P < 0.005.

Figure 4 presents heatmaps for Spearman correlations with respect to each PFT measure and scenario when evaluating SPECT perfusion maps. The first two columns for raw-valued images show fair to moderate correlations when the lungs are not discretized. The last two columns show a considerable increase in correlation strength when discretizing into Voronoi subvolumes. In particular, the strongest observed correlation (Spearman *ρ* = −0.811) occurs for FEV1/FVC when the lungs are analyzed together using a Voronoi discretization of radius *r* = 23, generating approximately 134 total subvolumes. Within each subvolume, taking the 75th percentile and then computing the fraction of subvolumes below 89.6% captures the most informative regional differences in perfusion. This combination of metrics highlights the subvolumes that are not among the very best-perfused regions. Since the threshold is high, most subvolumes will fall below it, so the resulting fraction captures the overall distribution of perfusion, which reflects how the majority of the lung is perfused, rather than only highlighting the rare, extremely low-function areas, like when studying ventilation maps.

As anticipated, performing descriptive statistics on the percentile-scaled SPECT images (bottom heatmap of figure 4) without Voronoi discretization yields poor correlations compared to the corresponding raw-valued perfusion maps. One of the strongest correlations of the percentile-scaled SPECT images (*ρ* = 0.789) is also found with FEV1/FVC data, but now with all cases being discretized to a radius of *r* = 74 (roughly 6 total subvolumes), taking the median percentile within each subvolume, computing the fraction of subvolumes below 53.8% for each lung, and then averaging the two lungs weighted by volume. Once again, this combination of metrics captures most of the general heterogeneity, while still highlighting moderately underperfused areas rather than only the extreme deficits. The largest improvement for these percentile perfusion correlations occurs for FVC (%), jumping from a negligible 0.079 to a substantial 0.590 in magnitude after discretizing. Correlations remain below 0.5 without discretization, but all elevate above 0.5 with discretization, highlighting the critical role of regional information. The heatmaps of figure 4 also depict an increase in correlation strength often times when LR lungs are treated separately, especially for percentile-scaled images. Comparison of the two rightmost columns reveals that different metric combinations yielding moderate correlations appear in each heatmap, with FVC (%) being the least correlated. Most correlation coefficients worsened (2 improved, 1 remain unchanged) when SPECT images were rescaled to percentiles, though the resulting correlations remained fair.

**Figure 5.**
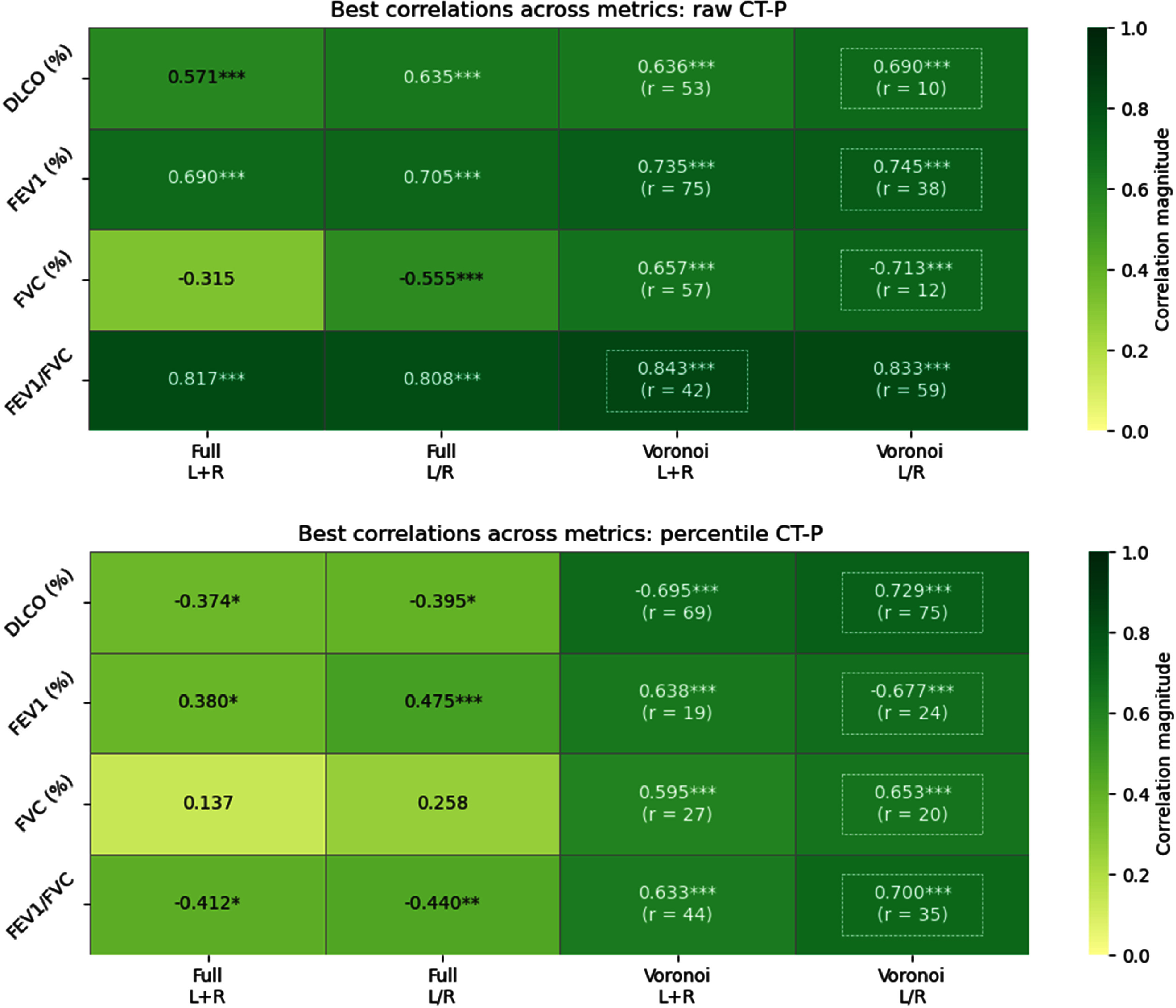
Spearman correlations for the best-performing hierarchical metrics on CT-P perfusion estimates with raw values (top) and percentiles (bottom). ‘Full’ denotes the perfusion map was not discretized, while ‘Voronoi’ denotes the map was discretized with the given radius. ‘L+R’ denotes the lungs are treated together, while ‘L/R’ denotes the lungs are treated separately. The strongest correlation for each PFT measure is highlighted by a white dashed box. Statistical significances: *P < 0.05, **P < 0.01, ***P < 0.005.

While CT-P estimates from 4DCT inhale/exhale phases have been reported to correlate fairly with SPECT perfusion (Castillo 2021), our internal dataset demonstrated a weaker averaged Spearman correlation of 0.292 ± 0.151 (95% CI: [0.241, 0.356]). Even so, the top heatmap of figure 5 shows exceptionally strong correlations between CT-P images of raw SPECT-like values and PFT measures, especially FEV1/FVC (*ρ* > 0.800) and regardless of discretization or LR splitting. Similar to figure 4, correlations distinctly improve in magnitude with Voronoi discretizations, most notably for CT-P percentile images. Correlations from discretized raw CT-P images ($0.636 < \rho < 0.843$) are generally higher than the discretized percentile images ($0.595 < \rho < 0.729$), but the latter continue to exhibit moderate correlations.

**Figure 6.**
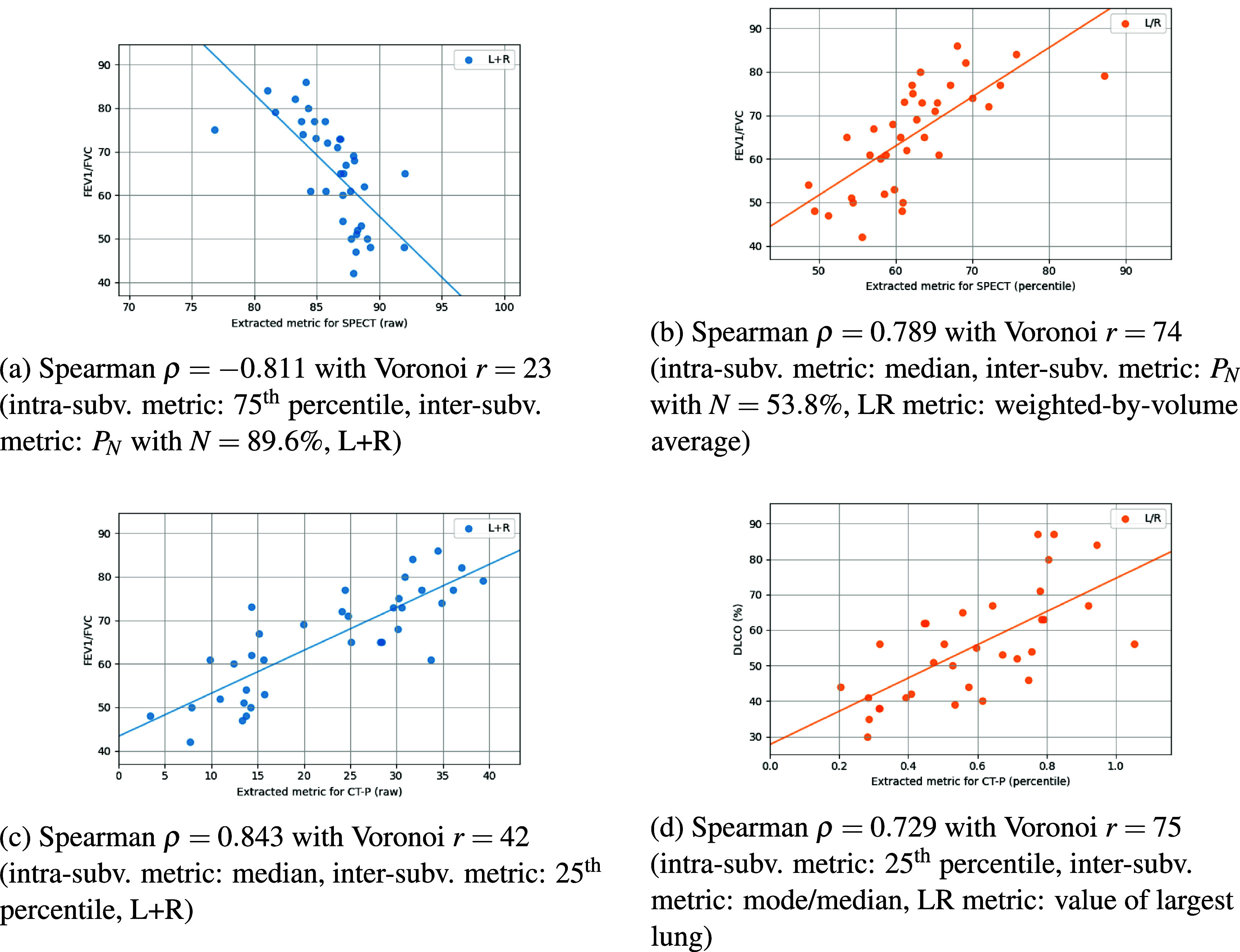
Scatter plots of the top performing hierarchal metrics for each of the four perfusion map types. The solid lines here indicate fitted trends.

For all four perfusion map types, discretization into Voronoi subvolumes consistently yielded higher correlated global statistics than without discretization. Tables 1 and 2 summarize the top 5 metrics for correlating raw and percentile SPECT and CT-P images, respectively, with DLCO (%), FEV1 (%), FVC (%), and FEV1/FVC. The top metrics in these tables correspond to the performances seen in figures 4 and 5. Across all PFT measures, metrics from raw SPECT images generally yielded stronger correlations than those from percentile-scaled images, particularly for FEV1/FVC and DLCO (%). Many of the best-performing metrics incorporated Voronoi discretization and the *P_N_* statistic, highlighting the importance of capturing local spatial heterogeneity. Notably, the dominant metric types varied across PFTs, with percentile-based values (median, 75th) driving correlations for DLCO (%) and FEV1/FVC, while measures such as CoV, mode/median, and skewness were more influential for FEV1 (%). The top-ranked SPECT metrics show stronger overall correlations with PFTs than CT-P, especially for DLCO (%) and FEV1/FVC, while CT-P achieves its highest correlations with FEV1/FVC but is weaker for DLCO (%). SPECT results are dominated by the cumulative volume measure *P_N_*, whereas CT-P relies more on global distribution statistics such as mean, median, and skewness. FVC (%) results are excluded hereafter due to their comparatively lower correlations and (subsequent) predictive power. As shown in bold in tables 1 and 2, the strongest correlated PFT measures were observed when the lungs were treated together (‘L+R’) and with raw-valued maps. In contrast, all top-performing metrics for percentile-scaled maps were found when the lungs were treated separately (‘L/R’). Figure 6 shows the scatter plots for the hierarchal metrics that yield the top correlations for each map type. Scatter plots for the best top hierarchal metrics per PFT measure can be found in figures S.1–S.4 in the supplementary data file.

**Table 1.** Top 5 ranked metrics correlating raw and percentile SPECT perfusion images with PFT measures. An empty ‘LR metric’ implies the lungs were treated together (‘L+R’). The strongest overall correlated PFT measure is bolded for each perfusion type.

		Spearman *ρ*	Voronoi *r*	Intra-subvol. metric	Inter-subvol. metric	LR metric
Raw images	DLCO (%)	−0.727	37	Median	${P_N \,(N = 77.5\%)}$	Max value
		−0.697	26	75th percentile	$P_N \,(N = 93.2\%)$	—
		−0.686	31	Median	$P_N \,(N = 72.1\%)$	—
		−0.683	23	75th percentile	$P_N \,(N = 92.0\%)$	—
		−0.678	23	Median	$P_N \,(N = 78.7\%)$	—
	FEV1 (%)	−0.730	70	75th percentile	$P_N \,(N = 40.5\%)$	—
		0.723	77	Median	25th percentile	Least perf.
		0.718	82	75th percentile	25th percentile	Least perf.
		0.716	43	75th percentile	25th percentile	Least perf.
		0.713	88	Mode/median	Mode/median	Min value
	FVC (%)	−0.636	45	75th percentile	$P_N \, (N = 10.7\%)$	—
		−0.634	53	CoV	Skewness	—
		−0.598	36	Max	$P_N \, (N = 20.7\%)$	—
		−0.594	65	Median	$P_N \, (N = 10.4\%)$	—
		−0.588	65	75th percentile	$P_N \, (N = 16.7\%)$	—
	**FEV1/FVC**	**−0.811**	**23**	**75th percentile**	$\boldsymbol{P_N \,(N = 89.6\%)}$	**—**
		−0.789	19	75th percentile	$P_N \,(N = 89.0\%)$	—
		−0.787	17	Median	$P_N \,(N = 77.5\%)$	—
		−0.787	34	75th percentile	$P_N \,(N = 86.3\%)$	—
		−0.784	17	75th percentile	$P_N \,(N = 90.8\%)$	—

Percentile images	DLCO (%)	0.647	19	Average	$P_N \,(N = 47.4\%)$	Smallest vol.
		0.619	19	Median	$P_N \,(N = 62.2\%)$	Smallest vol.
		−0.615	36	Average	Mode/median	Min value
		0.600	29	Median	$P_N \,(N = 70.6\%)$	Smallest vol.
		0.595	43	Median	$P_N \,(N = 59.2\%)$	Smallest vol.
	FEV1 (%)	0.789	35	Min	$P_N \,(N = 8.6\%)$	Perf.-weighted avg.
		0.734	74	Skewness	25th percentile	Min value
		−0.717	74	Median	75th percentile	Max value
		−0.715	38	CoV	Skewness	Smallest vol.
		0.709	32	Min	$P_N \,(N = 28.5\%)$	Perf.-weighted avg.
	FVC (%)	−0.590	90	Skewness	$P_N \, (N = 39.3\%)$	Largest vol.
		0.589	24	Median	$P_N \, (N = 7.4\%)$	Smallest vol.
		0.575	12	75th percentile	$P_N \,(N = 5.3\%)$	Max value
		0.562	24	75th percentile	$P_N \,(N = 6.5\%)$	Smallest vol.
		0.554	68	CoV	75th percentile	Min value
	**FEV1/FVC**	**0.789**	**74**	**Median**	$\boldsymbol{P_N \,(N = 53.8\%)}$	**Vol.-weighted avg.**
		0.752	74	25th percentile	$P_N \,(N = 26.4\%)$	Perf.-weighted avg.
		−0.738	76	Mode/median	CoV	Average
		−0.736	87	Mode/median	75th percentile	Min value
		0.734	87	Mode/median	$P_N \,(N = 5.3\%)$	Average

**Table 2.** Top 5 ranked metrics correlating raw and percentile CT-P images with PFT measures. An empty ‘LR metric’ implies the lungs were treated together (‘L+R’). The strongest overall correlated PFT measure is bolded for each perfusion type.

		Spearman *ρ*	Voronoi *r*	Intra-subvol. metric	Inter-subvol. metric	LR metric
Raw images	DLCO (%)	0.690	10	25th percentile	25th percentile	Largest vol.
		0.686	49	25th percentile	25th percentile	Largest vol.
		−0.686	15	Median	$P_N \,(N = 18.2\%)$	Largest vol.
		0.685	52	25th percentile	25th percentile	Largest vol.
		0.682	85	25th percentile	Mode/median	Vol.-weighted avg.
	FEV1 (%)	0.745	38	Average	Median	Min value
		0.739	36	Average	25th percentile	Min value
		0.739	74	Average	75th percentile	Min value
		0.739	84	25th percentile	Median	Min value
		0.739	32	Average	25th percentile	Min value
	FVC (%)	−0.713	12	Average	$P_N \,(N = 80.6\%)$	Most perf.
		−0.701	12	25th percentile	$P_N \,(N = 75.4\%)$	Most perf.
		−0.690	23	Average	$P_N \,(N = 85.4\%)$	Most perf.
		−0.657	12	Median	$P_N \,(N = 80.9\%)$	Most perf.
		−0.649	19	Average	$P_N \,(N = 74.8\%)$	Most perf.
	**FEV1/FVC**	**0.843**	**42**	**Median**	**25th percentile**	**—**
		0.842	34	Average	25th percentile	—
		0.841	12	25th percentile	25th percentile	—
		0.839	55	Average	25th percentile	—
		0.838	36	75th percentile	25th percentile	—

Percentile images	**DLCO (%)**	**0.729**	**75**	**25th percentile**	**Mode/median**	**Largest vol.**
		0.729	69	Median	$P_N \,(N = 24.3\%)$	Vol.-weighted avg.
		−0.723	64	25th percentile	$P_N \,(N = 7.4\%)$	Largest vol.
		−0.719	56	Median	$P_N \,(N = 14.9\%)$	Largest vol.
		>0.715	47	Average	$P_N \,(N = 56.8\%)$	Average
	FEV1 (%)	−0.677	24	Median	75th percentile	Max value
		−0.670	52	Skewness	$P_N \,(N = 44.4\%)$	Largest vol.
		−0.66	51	75th percentile	Mode/Median	Max value
		0.660	19	Min	$P_N \,(N = 79.0\%)$	Vol.-weighted avg.
		0.659	20	Median	$P_N \,(N = 30.6\%)$	Min value
	FVC (%)	0.653	20	Average	$P_N \,(N = 31.8\%)$	Min value
		−0.646	23	Median	Median	Max value
		0.644	23	75th percentile	$P_N \,(N = 51.7\%)$	Max value
		0.643	19	Max	$P_N \,(N = 36.9\%)$	Max value
		0.633	36	Median	$P_N \,(N = 93.8\%)$	Min value
	FEV1/FVC	0.700	35	Average	$P_N \,(N = 93.5\%)$	Max value
		−0.697	52	Skewness	$P_N \,(N = 41.7\%)$	Max value
		0.696	90	Mode/median	$P_N \,(N = 5.0\%)$	Largest vol.
		−0.689	86	Mode/median	CoV	Min value
		0.684	86	25th percentile	$P_N \,(N = 24.3\%)$	Perf.-weighted avg.

Although no formal visual assessment of perfusion defects was performed, we applied standard quantitative metrics intended to approximate visual evaluation, such as *P_N_*. However, the consistently stronger correlations observed for discretized metrics (see figures 4, 5 and tables 1, 2) compared with non-discretized measures suggest that visual assessment or conventional global metrics alone do not fully capture perfusion heterogeneity and that structured spatial discretization is necessary.

### 3.2. Logistic regression

For each of the four perfusion types, logistic regression models yield robust performances in predicting lung function abnormalities. Stepwise inclusion of the top-performing metrics (see figures S.5–S.8) at times induces collinearities, meaning that successive metrics contribute overlapping information to the model that noticeably diminish performance. The correlation matrices of figures S.9–S.12 depict generally strong pairwise correlations. In addition, standard VIF analysis from tables S.1 and S.2 displays very high multicollinearity for all covariates regardless of perfusion map type, so we compute a modified cross-group VIF to remove this inherent collinearity for covariates within the same PFT test parameter. These tables show that covariates from different PFT measures have low to moderate multicollinearities and suggest that covariates from various PFT measures can be combined to build the optimal predictive model.

**Figure 7.**
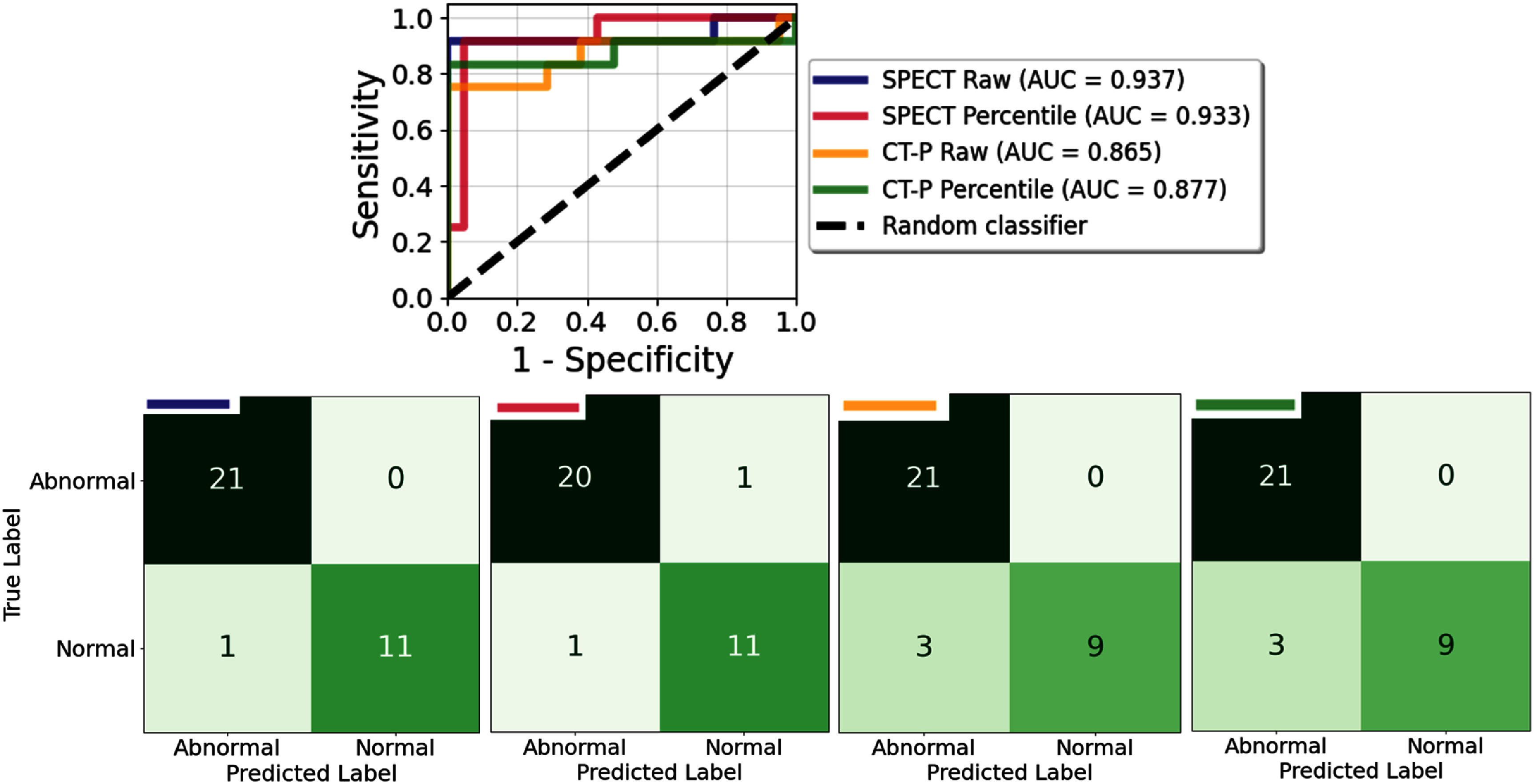
ROC-AUC results and confusion matrices for logistic regression models that use the best combination of all metrics as inputs for each perfusion map type.

A brute-force search of all possible combinations reveals the best predictive models for each perfusion map type presented in tables 3 and 4, listing the AUC scores, *F*1 scores, and accuracies for each model, along with the covariates and their regression coefficients. The regression models from SPECT images required noticeably more covariates (between 7 and 9) than those from CT-P images (between 3 and 5). We find excellent performance for raw and percentile SPECT images, reaching AUC scores of 0.937 and 0.933, respectively, while a slight drop to 0.865 and 0.877 for both raw and percentile CT-P images, respectively. As expected, given that SPECT serves as the reference standard for perfusion assessment, regression models using raw SPECT global statistics as covariates outperformed those derived from all other perfusion map types across the three performance metrics, with the percentile-scaled SPECT model demonstrating comparably strong performance as the second best. Figure 7 depicts ROC-AUC results and confusion matrices for these models, mitigating exceptionally well false positives (incorrectly predicting a patient has normal lung function).

**Table 3.** Performances of the best logistic regression models for SPECT images, along with the covariates and their coefficients, where the coefficients reflect variable importance. Mean ± standard deviations are given, relative to leave-one-out validation. The covariates here are previously defined in table 1.

	Raw images	Percentile images
AUC	0.937	0.933
*F*1 score	0.957	0.917
Accuracy	0.970	0.939

Covariates (coefficients)	2nd DLCO $(-1.269 \pm 0.039)$	3rd DLCO $(-1.105 \pm 0.052)$
	1st DLCO $(-1.204 \pm 0.042)$	2nd DLCO $(0.806 \pm 0.073)$
	4th FEV1 $(0.715 \pm 0.062)$	4th DLCO $(0.854 \pm 0.067)$
	5th FEV1 $(-0.644 \pm 0.099)$	2nd FEV1 $(-0.860 \pm 0.139)$
	5th FEV1/FVC $(-0.509 \pm 0.061)$	4th FEV1 $(-0.666 \pm 0.059)$
	5th DLCO $(-0.313 \pm 0.059)$	3rd FEV1/FVC $(-0.467 \pm 0.050)$
	3rd DLCO $(0.078 \pm 0.051)$	1st FEV1/FVC $(0.615 \pm 0.067)$
	—	2nd FEV1/FVC $(0.349 \pm 0.061)$
	—	1st FEV1 $(-0.253 \pm 0.045)$

Bias	$-0.970 \pm 0.110$	$-1.270 \pm 0.096$

**Table 4.** Performances of the best logistic regression models for CT-P images, along with the covariates and their coefficients, where the coefficients reflect variable importance. Mean ± standard deviations are given, relative to leave-one-out validation. The covariates here are previously defined in table 2.

	Raw images	Percentile images
AUC	0.865	0.877
*F*1 score	0.857	0.857
Accuracy	0.909	0.909

Covariates (coefficients)	5th DLCO $(0.863 \pm 0.081)$	5th DLCO $(1.289 \pm 0.080)$
	2nd DLCO $(0.760 \pm 0.087)$	4th FEV1/FVC $(1.224 \pm 0.059)$
	4th FEV1/FVC $(0.599 \pm 0.065)$	3rd FEV1/FVC $(-0.602 \pm 0.126)$
	—	2nd FEV1/FVC $(-0.527 \pm 0.051)$
	—	1st FEV1 $(-0.435 \pm 0.081)$

Bias	$-0.948 \pm 0.113$	$-0.922 \pm 0.143$

## 4. Discussion

We developed a three-level metric to evaluate perfusion images, consisting of intra-subvolume, inter-subvolume, and left-right stages. Each level compares distinct information about lung function. The intra-subvolume stage operates on individual Voronoi subregions of the lung, capturing the gradient of perfusion information within each region, producing a value that represents localized lung function at every subvolume, independent of whether the images are raw-valued or percentile-scaled. The inter-subvolume stage then combines information across the lung using descriptive statistics, yielding values that describe the function of each lung side. Because the left and right lungs often differ in overall health and function, one lung can disproportionately influence global PFT measurements. For example, in cases of unilateral disease such as lung cancer, one lung may be severely affected while the contralateral lung remains healthy. Equal weighting of both lungs could therefore produce global PFT values that underestimate the severity observed in the affected lung. To address this, the LR metrics allow differential weighting of the two lungs, generating a final statistic that best reflects global lung function in a manner consistent with conventional PFT measurements.

Discretizing the lungs into Voronoi subvolumes substantially improves the correlation of perfusion metrics with PFT measures, especially for DLCO (%) and FVC (%), clearly seen in the heatmaps of figures 4 and 5. Percentile images pose a challenge, because scaling removes absolute quantitative information and typical global descriptive statistics perform poorly. Voronoi discretization, however, captures local perfusion gradients, preserving clinically meaningful heterogeneity, particularly for FEV1/FVC based on our examinations, despite the cases where individual lungs are treated independently and percentile-scaled. The results in tables 1 and 2 highlight the value of incorporating local spatial information when linking perfusion maps to pulmonary function. Metrics derived from Voronoi-discretized subvolumes, particularly those using *P_N_* or intra-subvolume percentile statistics, consistently yielded the strongest correlations with PFT measures, underscoring the importance of capturing regional heterogeneity rather than relying on global averages alone. Interestingly, the specific metrics that performed best varied across PFT measures, suggesting that different aspects of perfusion variability, such as underperfused regions, distribution asymmetry, or local extremes, may drive different functional deficits. These findings support the utility of spatially-resolved analyses for improving the physiological interpretability of perfusion imaging.

Although DLCO is classically regarded as the physiologic measure most directly linked to pulmonary perfusion, our findings revealed stronger correlations between SPECT perfusion and spirometric measures. DLCO reflects gas diffusion across the alveolar-capillary membrane and can be reduced by loss of alveolar surface area, fibrotic thickening, or vascular injury-all processes closely tied to pulmonary perfusion (Goldin and Cascella 2025). Based on this physiology, we anticipated that the proposed three-level metrics would correlate most strongly with DLCO (%). However, our findings showed that SPECT perfusion correlated better with FEV1 (%) and FEV1/FVC. One possible explanation is that our cohort consists primarily of lung cancer patients, who often exhibit obstructive physiology due to tumor-related airway compression, radiation-induced narrowing, or bronchial changes. In such cases, DLCO may be reduced globally without clear spatial heterogeneity, limiting its alignment with regional perfusion patterns. By contrast, SPECT directly depicts regional blood flow, and its heterogeneous patterns may not capture the same diffusion-limiting mechanisms measured by DLCO. Furthermore, DLCO is highly sensitive to anemia, a common comorbidity in cancer patients, adding further variability (Pirker *et al* 2003, Kang *et al* 2020). In contrast, FEV1 and FEV1/FVC primarily reflect airflow obstruction, particularly in regions affected by tumor burden or radiation-induced inflammation. Because airflow limitation often coincides with reduced perfusion in lung cancer patients, these PFT measures demonstrated the strongest correlations with raw and percentile SPECT perfusion maps. Tables 1 and 2 also show that FVC (%) had some of the weakest correlations in both SPECT and CT-P images. We suspect the reason that we cannot extract the same information as other PFT parameters is due to the nature of what FVC measures. The FVC measures the amount of air an individual can forcibly inhale, which is heavily influenced by the size of the individual, making it much less indicative of lung function alone. For instance, an individual with smaller body size and normal lung function may exhibit the same FVC measurement as a larger individual with impaired lung function. The majority of the time, FVC is used either in longitudinal studies or in conjunction with other PFT parameters, like FEV1 in FEV1/FVC (Ponce *et al* 2025).

Moreover, across the 36 cases, SPECT and CT-P perfusion maps demonstrated only a modest Spearman correlation with one another (0.292 ± 0.151), despite each showing strong associations with PFT measures. This weak correspondence highlights the fundamentally different routes of acquiring the perfusion images: SPECT directly depicts radiotracer-based regional blood flow, whereas CT-P infers perfusion from volume and intensity variations within a non-contrast CT inhale/exhale pair. The ability of both modalities to independently correlate well with PFT suggests that CT-P may be capturing a distinct, yet physiologically meaningful, signal not represented in SPECT, potentially reflecting complementary aspects of pulmonary function.

CT-P outperformed SPECT with raw-valued maps when correlating with PFT, whereas SPECT achieved better results with percentile maps. Notably, CT-P’s strongest correlations shifted from FEV1/FVC (raw) to DLCO (%) (percentile), while SPECT consistently aligned with FEV1/FVC across both map types. For SPECT, optimal three-level metrics consistently employed *P_N_*, suggesting it is particularly effective for extracting lung-function signals from SPECT, whereas CT-P relied on a broader set of descriptive statistics. This distinction may explain why percentile CT-P metrics correlate more with DLCO (%), reflecting sensitivity to perfusion and gas-exchange capacity rather than airflow obstruction. Despite slight decreases in correlation when moving from raw to percentile images (2.7% decrease for SPECT and 13.5% decrease for CT-P in best correlations), the proposed three-level metrics maintained strong associations with PFTs and high predictive accuracy, underscoring its ability to capture clinically meaningful information from both raw and percentile perfusion maps.

After identifying metrics that correlate with PFT data, we next assessed their predictive power for classifying normal versus abnormal lung function. To ensure stable logistic regression results, we evaluated collinearity using pairwise comparisons and VIF. As expected, covariates derived from the top metrics for the same PFT parameter showed high collinearity (VIF $\gg$ 10) in tables S.1 and S.2, since they essentially capture the same underlying signal (Yoo *et al* 2014). To address this, we applied a cross-group VIF, comparing covariates only across different PFT parameters, revealing substantially lower multicollinearity and indicating that while metrics within a PFT group extract similar information, metrics across groups capture distinct and complementary aspects of lung function.

For raw SPECT images, the combined use of selected metrics was highly effective at distinguishing healthy from diseased patients, performing comparably to percentile SPECT and outperforming both raw and percentile CT-P. All analyses were conducted using a simple logistic regression model, yet the high AUC scores (between 0.865 and 0.937) and accuracies (between 0.909 and 0.970) for all four models highlight that these metrics isolate predictive information on lung function in a transparent and interpretable way. While predictive performance did not perfectly mirror correlations with PFT measures (see confusion matrices in figures S.9–S.12), most models followed the expected trend of lower accuracy with weaker three-level metric correlation. The notable exception was FEV1 (%) for both raw and percentile SPECT perfusion maps, where figures S.5 and S.6 show that the best predictive performance arose from the fifth-best and fourth-best correlating metrics, respectively, rather than the top-ranked one, suggesting the effect is intrinsic to the FEV1 (%) measure itself. Since FEV1 reflects the volume of air exhaled in 1 s and does not rely on a complete exhalation, unlike other the spirometric PFT measures, this unique physiological basis may explain why correlation strength and predictive power diverge in this case. On a similar note, in CT-V correlation studies (ventilation estimates derived from CT-based methods like CT-P), Brennan (2015) reported AUC scores of 0.64–0.72 for predicting abnormal lung function using CoV as the covariate. The results of this study suggest that correlations could be improved by discretizing the ventilation image, raw-valued or not, and applying hierarchical metrics as demonstrated here.

The use of three-level metrics creates an opportunity to train machine learning models with richer supervision. Although paired non-contrast CT and perfusion datasets are limited, large numbers of CT images and corresponding PFT measurements are readily available, owing to their low cost and noninvasive nature. Our three-level metric bridges this gap by enabling perfusion-derived predictions to be translated into PFT-like values, which can then be validated against ground-truth PFTs. This approach effectively expands the amount of usable training data, even in the absence of paired perfusion scans, and supports the development of more robust lung perfusion prediction models. Importantly, the hierarchal system performs well on percentile-based perfusion images, which is encouraging for AI models, such as the state-of-the-art approaches, that predict percentiles for individual lungs (Porter *et al* 2021, Liu *et al* 2025). Together, this framework increases data accessibility and facilitates the training and validation of clinically useful models.

In addition, this hierarchical pipeline is not intended to replace clinical assessments such as PFTs nor serve as a stand-alone lung function assessment tool. Rather, CT-derived perfusion provides a spatially resolved visualization of lung function, whereas PFTs summarize function with a single global value. Our study addresses the key question of whether CT-derived perfusion can be trusted as a surrogate by demonstrating strong correlations with PFTs and discrimination between normal and abnormal lung function. In this way, our work supports the reliability of CT-derived perfusion as a complementary, noninvasive functional imaging modality whose global perfusion scores are informed by the specific characteristics of the perfusion map. Our study has potential clinical impact by increasing a clinician’s confidence in perfusion images, particularly for CT-derived or AI-generated estimates. For example, if a clinician has access to a perfusion map without accompanying PFT data, they could apply the recommended Voronoi radii and metric combinations from tables 1 and 2 to extract global perfusion scores. These scores could then be input into our trained logistic regression model to predict the patient’s lung function or to corroborate visible symptoms, thereby enhancing trust in the perfusion estimate and providing a more interpretable visualization of regional lung function. Another scenario arises when a clinician has access to both a perfusion map and PFT data. In this case, the clinician could follow the same steps as in the previous example, while also comparing the magnitude of the derived perfusion scores with the PFT measurements. This comparison not only reinforces confidence in the perfusion estimate, but can also help identify patients at higher risk, whose scores and symptoms may warrant more urgent clinical attention. Lastly, if the best combination of metrics is applied to a perfusion estimate and the resulting global perfusion score is inconsistent with the patient’s symptoms or available PFT data, the clinician should not rely on the perfusion estimate alone. In such cases, alternative diagnostic imaging (e.g. SPECT) or the PFT data should guide clinical decision-making.

While our findings demonstrate the utility of the proposed three-level metrics, there are important limitations that warrant consideration and further investigation. Our study was conducted solely from one internal dataset with 36 cases from one institution following a specific protocol (see section 1), where patient cancer stages and treatments varied (radiotherapy, tumor resection, etc). Future studies will include more diversity in patients and acquisition protocols to assess the generalizability and stability of the top-performing metrics derived in this study. Secondly, for certain PFT measures that are less sensitive to perfusion characteristics, such as FVC (%), the hierarchical metrics tend to compress data points toward the extremes (e.g. near 0 or 100 on the metric axis in scatter plots), resulting in visually clustered distributions and reduced influence of these points on the overall correlation. This behavior is exhibited in 5 of the top 16 metrics across all four perfusion map types and four PFT measures, see figures S.1(b), (c), S.2(b), (c) and S.4(d) These cases warrant closer examination to understand the underlying cause and to develop strategies to mitigate such effects. While the current pipeline aims to maximize correlation strength, future iterations should incorporate mechanisms to account for and prevent these artificial clustering behaviors. In addition, there is the issue of artifacts, which are nearly unavoidable when using free-breathing 4DCT scans. These artifacts produce discontinuities in the lung volumes, hindering the clinical reliability of a scan and damaging the validity of the deformable image registration algorithm used to compute perfusion via CT-P. Artifacts also likely contributed to the low correlation observed between CT-P predictions and SPECT images, indicating the need to refine parameter choices for our cases within the CT-P method itself. Lastly, future studies will focus on region-specific discretization of the lungs to better capture spatial perfusion patterns, for example by using segmentation masks of the pulmonary arteries or quadtree-like dependence on image intensities to define perfusion territories or Voronoi seed points.

## 5. Conclusion

We developed an innovative hierarchical metric where perfusion functional maps are evaluated in three stages: intra-subvolume, inter-subvolume, and left-right, regardless if the maps consist of raw SPECT-like values or normalized to percentiles. Following an extensive computational search, we effectively are able to condense a 3D perfusion image down to one global score that can be used to strongly correlate with PFT data and accurately predict pulmonary function. Discretizing the lungs into spatially-coherent Voronoi subvolumes allows metrics to reflect clinically relevant heterogeneity that would be missed by whole-lung analyses. Importantly, the metric performs robustly on percentile-based perfusion images, which is highly relevant for validating state-of-the-art AI models and for guiding future approaches that predict lung-specific percentile maps. Based on our findings (which warrant further investigation to assess their generalizability), we recommend users to follow the metrics in tables 1 and 2 to achieve the strongest correlations with the available PFT data. This hierarchical framework is not intended to replace PFTs, but rather to complement them by providing spatially resolved perfusion information that yields global scores strongly correlated with lung function and capable of discriminating normal from abnormal physiology. By improving the interpretability and reliability of CT-derived or AI-based perfusion estimates, the approach can increase clinician confidence while appropriately deferring to PFTs or alternative imaging when discrepancies arise. Overall, our approach highlights the growing potential for diagnostic tools that leverage a single perfusion image—whether raw or percentile-scaled, SPECT or CT-derived—to discern pulmonary pathology effectively and noninvasively.

## Data Availability

The data cannot be made publicly available upon publication due to legal restrictions preventing unrestricted public distribution. The data that support the findings of this study are available upon reasonable request from the authors.
